# The balance between antibiotic resistance and fitness/virulence in *Pseudomonas aeruginosa*: an update on basic knowledge and fundamental research

**DOI:** 10.3389/fmicb.2023.1270999

**Published:** 2023-09-28

**Authors:** Elena Jordana-Lluch, Isabel Mª Barceló, María Escobar-Salom, Miguel A. Estévez, Laura Zamorano, Silvia Gómez-Zorrilla, Elena Sendra, Antonio Oliver, Carlos Juan

**Affiliations:** ^1^Research Unit, University Hospital Son Espases-Health Research Institute of the Balearic Islands (IdISBa), Palma, Spain; ^2^Department of Microbiology, University Hospital Son Espases, Palma, Spain; ^3^Centro de Investigación Biomédica en Red, Enfermedades Infecciosas (CIBERINFEC), Madrid, Spain; ^4^Infectious Diseases Service, Hospital del Mar, Hospital del Mar Research Institute, Barcelona, Spain; ^5^Universitat Pompeu Fabra (UPF), Universitat Autònoma de Barcelóna (UAB), Barcelona, Spain

**Keywords:** *Pseudomonas aeruginosa*, antibiotic resistance, horizontal β-lactamases, AmpC, efflux pumps, fitness, virulence, biological cost

## Abstract

The interplay between antibiotic resistance and bacterial fitness/virulence has attracted the interest of researchers for decades because of its therapeutic implications, since it is classically assumed that resistance usually entails certain biological costs. Reviews on this topic revise the published data from a general point of view, including studies based on clinical strains or *in vitro*-evolved mutants in which the resistance phenotype is seen as a final outcome, i.e., a combination of mechanisms. However, a review analyzing the resistance/fitness balance from the basic research perspective, compiling studies in which the different resistance pathways and respective biological costs are individually approached, was missing. Here we cover this gap, specifically focusing on *Pseudomonas aeruginosa,* a pathogen that stands out because of its extraordinary capacity for resistance development and for which a considerable number of recent and particular data on the interplay with fitness/virulence have been released. The revised information, split into horizontally-acquired vs. mutation-driven resistance, suggests a great complexity and even controversy in the resistance-fitness/virulence balance in the acute infection context, with results ranging from high costs linked to certain pathways to others that are seemingly cost-free or even cases of resistance mechanisms contributing to increased pathogenic capacities. The elusive mechanistic basis for some enigmatic data, knowledge gaps, and possibilities for therapeutic exploitation are discussed. The information gathered suggests that resistance-fitness/virulence interplay may be a source of potential antipseudomonal targets and thus, this review poses the elementary first step for the future development of these strategies harnessing certain resistance-associated biological burdens.

## 1. Introduction

The fitness (also known as biological success) of a bacterial species is its capacity to thrive within a given context defined by nutrient availability, physicochemical conditions, presence of antibiotics, etc. (Botelho et al., 2019). Evidently, when considering a pathogen microorganism, one feature intimately linked to fitness is virulence, since the greater the ability to infect an individual through virulence factors and strategies to evade immunity, the more chances it will have to survive and multiply (Beceiro et al., 2013). Therefore, although fitness has been classically quantified through growth rate and inter-bacterial competitiveness, these parameters also inseparably impact the pathogenic capacity of the bacterium: in general, the slower it grows inside the host, the less virulent it will be and vice versa, at least in acute infections (Andersson, 2006; Andersson and Hughes, 2010; Melnyk et al., 2015). Conversely, when considering chronic infections such as those typically appearing in the lungs of patients with cystic fibrosis, selection for slow growth and reduced virulence often seems to be the hallmark of successfully adapted strains and hence, the analysis of fitness is not that simple in this case (Høiby et al., 2010; Lorè et al., 2012; Malhotra et al., 2019). Therefore, in the present review we will focus on the interplay between resistance and fitness/virulence in the classic acute infection context, and appealing to a *sensu lato* conception, we will indistinctly refer to virulence or fitness because of their abovementioned close linkage although we know that, strictly speaking, they are not equivalent terms. Accordingly, the concepts of virulence attenuation and biological cost (or burden) will also be used as synonymous. Moreover, as shown below, not only growth but also other specific virulence-related parameters (motility, production of toxins, pigments and/or proteases, cytotoxicity, biofilm formation, etc.) (Gallant et al., 2005; Deptuła and Gospodarek, 2010; Stickland et al., 2010; Pérez-Gallego et al., 2016; Schroeder et al., 2017) are altered in association with certain resistance mechanisms, which supports our inclusive use of both fitness and virulence concepts.

Although there is a classic idea advocating that acquired antibiotic resistance often entails an associated biological cost, mostly in terms of reduced growth rate and competitiveness in assays mating susceptible vs. resistant strains, this assumption is far from being so straightforward in reality (Beceiro et al., 2013; Melnyk et al., 2015; Vogwill and MacLean, 2015; Melnyk et al., 2017). This notion is based on the fact that typical mutation-driven resistance mechanisms may have important impacts on relevant biological processes, which end up affecting bacterial growth and/or pathogenic behavior (Beceiro et al., 2013): (i) mutations in antibiotic targets that reduce the affinity of the drug but also impair the efficiency of the original protein; (ii) overexpression of β-lactamases entailing high energy costs because of the increased amount of protein produced. Additionally, the β–lactamase enzymatic activity itself and/or the mechanism of hyperproduction, potentially causing alterations in the peptidoglycan structure and/or its metabolism, could hinder cell viability; (iii) efflux pumps hyperexpression, which in parallel to antibiotics may excessively extrude other compounds needed for an effective pathogenic performance, such as quorum-sensing signals; (iv) porins loss, causing a reduced influx of the antibiotic but potentially also of other substances that could contribute to efficient growth. Additionally, certain porins (e.g., OprF) have been described to take part in specific pathogenesis-related events such as host cell adhesion, invasiveness, and biofilm formation; thus, these parameters would be altered if the porin is disrupted (Beceiro et al., 2013; Fernando and Kumar, 2013; Chevalier et al., 2017).

Meanwhile, regarding horizontally-acquired resistance, the fact of producing an external protein such as a β-lactamase in high amounts (or even large groups of other proteins codified in the same carrier mobile genetic element) may entail additional energy burdens and/or collateral effects on metabolism, which could impair growth (Beceiro et al., 2013; Barceló et al., 2022a). Interestingly, it has been proposed that the biological cost associated with horizontally-acquired resistance is overall lower than that of the mutation-driven mechanisms, that usually have greater impacts on bacterial biology through the abovementioned phenomena (Vogwill and MacLean, 2015).

Either way, following the aforementioned simplified conception of the interplay between resistance and fitness, susceptible strains would be expected to outcompete resistant ones if antibiotic selective pressure was eliminated, as has been experimentally demonstrated (Vogwill and MacLean, 2015). However, as will be shown throughout this review there is also a considerable number of examples in which the allegedly typical burden associated with resistance is not found at all, and even some cases of increased virulence linked to certain resistance pathways (Aoki et al., 2004; Gallant et al., 2005; Roux et al., 2015; Guillard et al., 2016; Jorth et al., 2017; Cervoni et al., 2021; Barceló et al., 2022a,b). The basis underlying these phenomena has been explained by different facts, i.e., (i) horizontally-acquired and mutation-driven resistance mechanisms that appear to be cost-free because of their intrinsic nature and features; (ii) genetic linkage or co-selection between resistance and other markers (e.g., presence of virulence factors encoded in the same plasmid harboring the resistance determinant); (iii) the selection of compensatory mutations that specifically reduce the initial burden associated with the particular resistance mechanism, and/or mutations that increase general fitness irrespectively of antibiotic resistance. In this last regard, the biological cost associated with certain resistance phenotypes in *Pseudomonas aeruginosa* has been shown to be alleviated in hypermutator backgrounds, likely due to an increased capacity to acquire cost-compensatory mutations compared to non-hypermutator strains (Perron et al., 2010; Cabot et al., 2014, 2016). All these circumstances would thus underlie the phenomenon of perpetuation of resistance mechanisms occurring even in the absence of antibiotic pressure (Andersson, 2006; Andersson and Hughes, 2010; Schulz Zur Wiesch et al., 2010; Melnyk et al., 2015). However, it has also been proposed that this outcome may be overestimated in laboratory conditions, in which case, compensatory adaptation would not be as effective *in vivo*, a circumstance that should be delved into for the development of therapies (MacLean and Vogwill, 2014; McLean et al., 2019; Hernando-Amado et al., 2022). Therefore, understanding the compensation mechanisms of resistance-associated fitness costs is a topic of great interest because of its potential therapeutic implications. In this sense, some papers on the topic using *P. aeruginosa* rifampicin resistance as a model revealed different typologies of compensatory mutations appearing in response to low vs. high costs associated with the resistant phenotype, as well as the existence of various epistatic interactions finally determining the associated burden depending on genetic background and external physicochemical conditions (Hall and MacLean, 2011; Qi et al., 2016; Vogwill et al., 2016; Gifford et al., 2016a,b; Souque et al., 2023). At any rate, an essential first step to fully understanding compensatory evolution (which by the way seems an insufficiently studied field at least in relevant pathogen species) is to completely dissect the resistance vs. fitness/virulence interplay from a basic and fundamental research perspective, the goal addressed in this review.

Altogether these facts suggest that the interplay between antibiotic resistance and fitness is a topic of extreme complexity in which many elements are involved. Thus, it has to be dissected by taking into account different factors, such as the specific resistance-causing pathway, scenario (*in vitro* vs. *in vivo*, laboratory conditions vs. clinical context), type of mechanistic basis (horizontally-acquired vs. mutation-driven), and, obviously, the bacterial species. A multitude of published studies in the field are available for different pathogens (Andersson, 2006; Andersson and Hughes, 2010; Beceiro et al., 2013; Melnyk et al., 2015, 2017; Guillard et al., 2016; Cepas and Soto, 2020), being an important part of the knowledge likely generalizable for a wide range of species. However, because of its outstanding importance as a human opportunistic pathogen, its multidrug-resistant nature, and the urgent need for the development of new therapeutic weapons (Bassetti et al., 2017; Reig et al., 2022), here we focus on the interplay between resistance and virulence in *P. aeruginosa* exclusively. We include classic works but also particular and/or recent results that have not been found in other species, and that therefore deserve an update. To make the analysis easier, the topic is split into two parts, respectively reviewing the basic research and fundamental knowledge about i) the impact of horizontally-acquired resistance on fitness/virulence; and ii) the burden associated with mutation-driven resistance mechanisms. An overview of the main findings on both subjects in *P. aeruginosa* is shown in Tables 1, 2, respectively.

**Table 1:** Overview of the main basic research concerning the interplay between horizontally-acquired antibiotic resistance and fitness/virulence in *P. aeruginosa.*

Horizontally-acquired enzyme	Main features	Assays/parameters measured	Effects on fitness/virulence	References
*bla*TEM-1	Narrow spectrum class A β-lactamase	*In vitro*: growth rate, attachment to surfaces, motility, biofilm formation	Impaired twitching motility, adherence and biofilm formation	Gallant et al. (2005)
*bla*OXA-3	Narrow spectrum class D β-lactamase	*Idem*	*Idem*	Gallant et al. (2005)
*bla*IMP-1	Class B carbapenemase	*In vitro*: cell culture invasion *In vivo*: murine bacteremia model	No attenuation of killing capacity in the murine model, although a slight decrease in the invasion capacity was seen.	Aoki et al. (2004)
*bla*VIM-1	Class B carbapenemase	*In vitro*: growth rates, motility, cell culture invasion, cytotoxicity, susceptibility to osmotic shock and lysozyme-mediated lysis *In vivo*: *G. mellonella* larvae killing model	Absence of significant impacts on *G. mellonella* killing capacity	Barceló et al. (2022a)
*bla*GES-1	Class A ESBL	*Idem*	*Idem*	Barceló et al. (2022a)
*bla*OXA-2	Narrow spectrum class D β-lactamase	*Idem*	Very slight virulence attenuation in *G. mellonella* model (minor increase in LD50 values: < 2-fold compared to wildtype)	Barceló et al. (2022a)
*bla*OXA-226	OXA-2-derived ESBL conferring resistance to CAZ/AVI and TOL/TAZ	*Idem*	Significant attenuation in *G. mellonella* model (notably increased LD50: *ca.* 3-fold)	Barceló et al. (2022a)
*bla*OXA-161	*Idem*	*Idem*	High attenuation in *G. mellonella* model (highly increased LD50: *ca.* 10-fold)	Barceló et al. (2022a)
*bla*OXA-539	*Idem*	*Idem*	Dramatic attenuation in *G. mellonella* model (extremely increased LD50: > 100-fold)	Barceló et al. (2022a)
*bla*FOX-4	*bla*FOX-3-related class C β-lactamase conferring resistance to CAZ/AVI and TOL/TAZ	*In vivo*: *G. mellonella* larvae killing model	Slight virulence attenuation in *G. mellonella* model (increase in LD50: < 3-fold)	Barceló et al. (2022b)
*bla*FOX-8	*bla*FOX-3-related class C β-lactamase with reduced cephalosporin hydrolytic capacity	*Idem*	*Idem*	Barceló et al. (2022b)
*bla*PSE-1 (CARB-2)	Class A penicillinase	*In vitro*: growth rates and production/activity of extracellular virulence factors (alginate, proteases, elastase and rhamnolipid) *In vivo*: murine model of acute respiratory infection	Virtually absent attenuation of virulence in mice and very minor effects on the rest of parameters.	Ramisse et al. (2000)
*mcr*-1	Phosphoethanolamine transferase driving to LPS modification and derived colistin resistance	*In vitro*: growth rates, competition assays, detergent sensitivity, permeability assays	Absence of biological burden: no effects on growth or cell envelope homeostasis	Cervoni et al. (2021)

ESBL, extended spectrum β-lactamase; CAZ/AVI, ceftazidime-avibactam; TOL/TAZ, ceftolozane/tazobactam; LD50, lethal dose 50; LPS, lipopolysaccharide.

**Table 2:** Overview of the main basic research published data regarding the interplay between mutation-driven antibiotic resistance and fitness/virulence in *P. aeruginosa*.

Mechanism	Antibiotics affected	Mutational targets	Assays/parameters measured	Overview of effects on fitness/virulence	References
AmpC hyperproduction	Antipseudomonal penicillins, 3rd and 4th generation cephalosporins, monobactams	Disruption of PGN metabolism-related genes (*ampD, ampDh2, ampDh3, dacB*)	*In vitro:* growth rates, competition assays, cytotoxicity, motility, susceptibility to PGN-lytic immune compounds, transcriptomics *In vivo: G. mellonella* larvae killing model	When hyperproduction was combined with PGN recycling blockade (triple *ampD-ampDh2-ampDh3* inactivation), all the mentioned parameters were significantly impaired. These results were supported by the hypo-expression of LasA, phospholipase C, type IV pili, LPS biosynthetic pathways, and T3SS components. No negative impacts for fitness/virulence detected for the most common natural mutational targets, such as *ampD* or *dacB* disruptions.	Moya et al. (2008), Pérez-Gallego et al. (2016), Torrens et al. (2017), and Barceló et al., (2022b)
		Transcriptional regulator AmpR: G154R	*In vivo: C. elegans* killing model	Found to contribute to reduced virulence in the *C. elegans* model.	Sánchez-Diener et al. (2017)
		Not determined	*In vitro*: growth rates and production/activity of extracellular virulence factors (alginate, proteases, elastase and rhamnolipid) *In vivo*: murine model of acute respiratory infection	When AmpC hyperproduction was combined with the production of the PSE-1 horizontal penicillinase, virulence on murine model was drastically attenuated, as well as the secretion of the measured virulence factors excepting alginate.	Ramisse et al. (2000)
Changes in AmpC hydrolytic spectrum	Additional resistance to TOL/TAZ and CAZ/AVI	Structural modification in AmpC itself: T96I, G183D, ΔG229-E247	*In vivo*: *G. mellonella* larvae killing model	No further virulence attenuation compared with wildtype AmpC hyperproduction.	Barceló et al. (2022b)
OprD loss	Carbapenems	Disruption of *oprD*	*In vitro*: growth rates, competition assays, biofilm formation, resistance to serum, cytotoxicity, production/activity of extracellular virulence factors *In vivo*: murine model of acute respiratory infection; murine model of gastrointestinal tract colonization and systemic dissemination	Controversial results depending on the study, ranging from slightly reduced virulence to hypervirulent behavior. Differences in the model strains used and the virulence-related parameters studied probably account for the contradictory outcomes.	Ramisse et al. (2000), Abdelraouf et al. (2011), Skurnik et al. (2013), and Roux et al. (2015)
MexAB-OprM hyperexpression	Quinolones, macrolides, tetracyclines, lincomycin, chloramphenicol, novobiocin, most β-lactams	*mexR*, *nalD*	*In vitro*: growth rates, competition assays, motility, production/activity of extracellular virulence factors, survival in water and on dry surfaces, biofilm formation, cell culture invasion *In vivo: C. elegans* killing model, murine models of acute respiratory infection and bacteremia	Controversial results depending on the study, ranging from reduced virulence (including a decreased production of proteases, elastase and pyocianin, impaired cell invasiveness and killing of nematodes/mice and reduced survival in water and on dry surfaces) to significantly increased virulence. Probably dependent on the strain and type of mutation (disruption vs. amino acid changes).	Evans et al. (1998), Hirakata et al. (2002), Sánchez et al. (2002), Abdelraouf et al. (2011), Jorth et al. (2017), and Vaillancourt et al. (2021)
MexCD-OprJ hyperexpression	Quinolones, macrolides, novobiocin, tetracyclines, chloramphenicol, cefepime	*nfxB*	*In vitro*: growth rates, competition assays, motility, production/activity of extracellular virulence factors, resistance to complement, survival in water and on dry surfaces, biofilm formation, cytotoxicity, T3SS activity, cell culture invasion, proteomics and metabolomics *In vivo: C. elegans* killing model, murine model of bacteremia	Clear attenuation of virulence reflected in: reduced nematode/mice killing, impaired cell invasiveness, growth, survival in water/dry surfaces, motility, production of siderophores, rhamnolipid, elastase, phospholipase C, and pyocyanin, impaired T3SS performance and resistance to complement as well as altered proteome-metabolome. Altogether, these data reflect a globally dysregulated physiology of the MexCD-OprJ hyperproducer mutants.	Hirakata et al. (2002), Sánchez et al. (2002), Linares et al. (2005), Jeannot et al. (2008), Stickland et al. (2010), and Martínez-Ramos et al. (2014)
MexEF-OprN hyperexpression	Chloramphenicol, quinolones, trimethoprim	*mexT, mexS, parRS*	*In vitro*: growth rates, competition assays, production/activity of extracellular virulence factors, cytotoxicity, T3SS activity, biofilm formation, motility *In vivo: D. discoideum, C. elegans*, acute respiratory infection murine model, rat pneumonia and colorectal anastomotic leak models	MexEF-OprN hyperproducers showed a reduced production of pyocianin, elastase, and rhamnolipids and impaired T3SS performance, swarming motility, and biofilm formation, which resulted in clear virulence attenuations in the different *in vivo* infection models. Accordingly, inactivation of the pump increased *P. aeruginosa* virulence *in vivo*, fact associated with improved growth, competitiveness, collagenase activity, rhamnolipid production, and swarming.	Köhler et al. (2001), Cosson et al. (2002), Linares et al. (2005), Lamarche and Déziel (2011), Olivares et al. (2012), Olivas et al. (2012), Wang et al. (2013), Luong et al. (2014), and Vaillancourt et al. (2021)
MexXY-OprM hyperexpression	Aminoglycosides, quinolones, cefepime, colistin	*mexZ, parRS*	*In vitro*: cell culture invasion assays, pyoverdine production *In vivo:* murine model of endogenous bacteremia	Reduced pyoverdine production associated with pump hyperexpression, but decreased invasiveness and lethality in mice caused by a MexXY-defective mutant.	Hirakata et al. (2002) and Ikarashi et al. (2021)
DNA gyrase/topoisomerase IV alterations	Quinolones	QRDR (*gyrA, gyrB, parC, parE*)	*In vitro*: growth rate, competition assays, biofilm formation	Virtually inexistent impact on fitness/virulence for low level resistance-causing mutations. High level resistance mutations associated with dramatic growth and competitiveness impairments, readily alleviated by compensatory mutations. Impacts on fitness/virulence reduced in ExoU^+^ strains.	Kugelberg et al. (2005), Agnello et al. (2016), Melnyk et al. (2017)
LPS modification (aminoarabino-sylation)	Colistin (polymyxin E)	Different mutations in two-component systems (*phoPQ, parRS, cprRS, pmrAB,* etc.) causing *arn* operon overexpression	*In vitro*: growth rate, biofilm formation, cell envelope integrity assays, resistance to serum *In vivo: G. mellonella* larvae killing model	Innocuous effects vs. increased serum resistance and biofilm formation plus attenuated virulence in *G. mellonella,* after 21 days of colistin exposure in morbidostat.	Lo Sciuto et al. (2020) and Javed et al. (2021)

PGN, peptidoglycan; CAZ/AVI, ceftazidime-avibactam; TOL/TAZ, ceftolozane/tazobactam; LPS, lipopolysaccharide; QRDR, Quinolone Resistance-Determining Regions; T3SS, type III secretion system.

## 2. Horizontally-acquired resistance

Virtually all the knowledge regarding the biological cost of horizontally-acquired resistance in *P. aeruginosa* deals with the incorporation of β-lactamases from any of Ambler’s classes, including extended spectrum β-lactamases (ESBLs) and carbapenemases (Yoon and Jeong, 2021). In *P. aeruginosa* these enzymes are usually encoded in class I integrons, which are the predominant platforms for acquisition of resistance markers and ulterior dissemination through transferable elements such as plasmids. Excellent reviews on the topic can be resourced for more information concerning these resistance-related vehicles and their mechanisms of horizontal transfer (Escudero et al., 2015; Zhao and Hu, 2015). Besides β-lactamases, it is usual that different aminoglycoside-modifying enzymes and even other less frequent determinants (e.g., fluoroquinolones and colistin resistance genes) are carried in integrons/plasmids (Chávez-Jacobo et al., 2018; Khan et al., 2020; Kocsis et al., 2021; Nitz et al., 2021), although little knowledge regarding their biological cost is available. An exception to this is a recent paper in which the burden of the colistin resistance-conferring gene *mcr-1*, more commonly found in *Enterobacteriaceae*, was assessed suggesting the worrisome absence of any associated fitness barrier for the spread of this determinant in *P. aeruginosa* (Cervoni et al., 2021; Table 1).

Returning to the balance between horizontal β-lactamase-dependent resistance and virulence in *P. aeruginosa*, the volume of published information is not as high as for other species (Marciano et al., 2007; Dubois et al., 2009; Cottell et al., 2012; Fernández A. et al., 2012; Cordeiro et al., 2014; Colquhoun et al., 2021, 2023; Rajer and Sandegren, 2022), but some interesting data are available. For instance, Gallant and colleagues showed that the production of the cloned enzymes TEM-1 and OXA-3 (an OXA-2-related variant) caused significant defects in twitching motility, adherence, and biofilm formation of *P. aeruginosa* without affecting its growth, which suggests a finely tuned pathogenic behavior modulation rather than a general loss of biological efficacy (Gallant et al., 2005). Meanwhile, the production of the penicillinase PSE-1 had no apparent effects on *P. aeruginosa* fitness/virulence according to Ramisse et al. (2000) (Table 1).

More recently, the influence of other acquired β-lactamases on *P. aeruginosa* biological success has been analyzed through different assays including the invertebrate *Galleria mellonella* infection model, revealing that the production of some widely disseminated enzymes such as the class B carbapenemase VIM-1, the class A ESBL GES-1, or the narrow spectrum OXA-2 caused virtually insignificant decreases in fitness/virulence (Barceló et al., 2022a; Table 1). In another recent study, the burden of producing two less frequent class C transferable enzymes related to FOX-3, namely FOX-4 (variant conferring resistance to ceftolozane/tazobactam and ceftazidime/avibactam) and FOX-8 (a derivative with reduced cephalosporin hydrolysis capacity) was approached, and a very minor impact on *P. aeruginosa* virulence was also demonstrated for both (Barceló et al., 2022b; Table 1). Returning to VIM-1, GES-1, and OXA-2 (Barceló et al., 2022a), the mentioned results were somehow to be expected, because if the biological burden associated to their expression were high, they would not be so usually detected in clinical samples (Oliver et al., 2015; Del Barrio-Tofiño et al., 2020; Kocsis et al., 2021). In this regard, some studies with different species have shown that plasmids harboring very diverse β-lactamases may carry other elements that balance their associated burden or even increase the pathogenic potential by codifying additional virulence factors (Harrison and Brockhurst, 2012; Carattoli, 2013; Tsang, 2017; Lee et al., 2020). However, this is not the case for the abovementioned VIM-1, GES-1, and OXA-2 in *P. aeruginosa*, since they were produced from multicopy vectors devoid of any extra virulence factor, demonstrating a very low burden of these enzymes by themselves (Barceló et al., 2022a). A similar result had previously been obtained with another cloned class B carbapenemase (IMP-1) that did not significantly alter *P. aeruginosa* virulence, supporting the wide dissemination of this metallo-β-lactamase in this and other species (Aoki et al., 2004, Pongchaikul and Mongkolsuk, 2022; Table 1). Interestingly, in the aforementioned work (Barceló et al., 2022a) it was shown that, in contrast to OXA-2, the production of its extended spectrum derivatives OXA-161, OXA-226, and especially OXA-539 entailed dramatic virulence attenuations. These were reflected in significant decreases in the *G. mellonella* larvae killing capacity, with lethal dose 50 values of the strains expressing the OXA-2 derivatives *ca.* several hundred-fold higher than those of the wild-type strain in some cases. Nevertheless, these phenotypes were seemingly not relying on the alteration of other fitness/virulence-related parameters such as growth rate, cytotoxicity, motility, or susceptibility to lysis (Barceló et al., 2022a; Table 1). In the light of these data, hypothesizing that the high biological cost may pose a barrier for a more usual selection/dissemination of certain enzymatic variants with increased hydrolytic capacity (in contrast with the originative enzymes), seems reasonable and likely applicable to at least some specific β-lactamase derivatives.

Although the exact mechanistic bases for the results exposed in these two previous paragraphs remain unclear, some explanations have been proposed. For instance, Gallant et al. hypothesized that β-lactamases from the A and D classes (TEM-1 and OXA-3), because of their relatedness to low molecular weight Penicillin Binding Proteins (PBPs), could be sequestering the peptidoglycan substrates of such enzymes in the periplasm. This fact would in turn alter the cell wall metabolism/architecture and, consequently, impair the abovementioned virulence-related parameters (Gallant et al., 2005). Interestingly, an intact enzymatic activity was shown to be necessary to cause the fitness cost associated with TEM-1 but not with OXA-3, suggesting a different mechanistic basis linked to each β-lactamase (Gallant et al., 2005). Closely related to this, the important virulence attenuation associated with the aforementioned ESBLs, OXA-161, OXA-226, and OXA-539 but not with their parental enzyme OXA-2, suggests certain involvement of the enzymes’ active sites, because if mutations change the hydrolytic spectrum of β-lactamases (from narrow to extended), why should they not be altering the effect on other substrates? Moreover, in this same work, it was shown that the higher the ceftazidime hydrolytic capacity of each variant (OXA539 > OXA-161 > OXA-226), the greater the virulence attenuation associated, reinforcing the idea of a starring role for the active site of the β–lactamases. In this sense, it was proposed that, given the common evolutionary origin of certain β-lactamases with endo/carboxy-peptidase PBPs, a residual peptidoglycan-degrading power could exist for the former, fact in which an alteration of cell wall metabolism could rely on to finally impair fitness/virulence (Chang et al., 1990; Bishop and Weiner, 1992; Damblon et al., 1995; Rhazi et al., 1999; Urbach et al., 2008; Fernández A. et al., 2012; Pratt, 2016; Barceló et al., 2022a,b). Obviously, this would be the case for the mentioned OXA-2-derived ESBLs but not for the originative enzyme, whose active site would remain unable to significantly cleave peptidoglycan. Whether the alleged residual activity of the mentioned ESBLs could affect the murein sacculus itself weakening its structure (Pérez-Gallego et al., 2016; Torrens et al., 2017; Barceló et al., 2022a,b) and/or potential peptidoglycan soluble fragments (muropeptides)-based signaling networks ultimately regulating pathogenic behavior (Folkesson et al., 2005; Escobar-Salom et al., 2023), remains to be elucidated.

Beyond these studies with specific enzymes, it is imperative to consider the typical event of association of *P. aeruginosa* high-risk clones (e.g., ST235, ST111, and ST175, among others) with different acquired β-lactamases, posing successful combinations of clonal lineages plus resistance determinants of epidemic dissemination. This fact clearly suggests that—despite having not been assessed in terms of their specific biological burdens—enzymes such as OXA-10, PER-1, NDM-1, KPC-2, VIM-2, and certain GES-type ESBLs/carbapenemases among many others, probably do not involve high costs for *P. aeruginosa* because if this were the case, their appearance would not be that frequent (Oliver et al., 2015; Del Barrio-Tofiño et al., 2020; Kocsis et al., 2021). On the other hand, it is not possible to fully rule out the well-known phenomenon of certain plasmids encoding features that alleviate the biological cost of β-lactamases contributing to this outcome (Harrison and Brockhurst, 2012; Carattoli, 2013; Tsang, 2017; Kottara et al., 2018; Pongchaikul and Mongkolsuk, 2022), although, to our knowledge, this fact has not been specifically demonstrated in *P. aeruginosa*. This contrasts with other species such as *K. pneumoniae*, in which the phenomenon seems to be frequent (Gomez-Simmonds and Uhlemann, 2017; Jia et al., 2022). Finally, although a very specific case of a natural plasmid harboring two widely detected β-lactamases (OXA-10 and VIM-2) entailing high biological costs when conjugated to *P. aeruginosa* has been recently described (Li et al., 2023), this does not seem to be a common occurrence at all and could probably be related to intrinsic features of the starring plasmid yet to be determined. Considering all these facts, the idea of a relatively low cost associated with most horizontal β-lactamases seems more than reasonable. The influence of the species in this outcome is obviously also important, being *P. aeruginosa* a very good carrier of enzymes in contrast to other pathogens for which the acquisition of specific horizontal β-lactamases seems to entail a significant cost, such as the particular case of *Salmonella enterica* serovar Typhimurium producing an *Enterobacter cloacae*-derived AmpC or a VIM-2 carbapenemase (Morosini et al., 2000; Cordeiro et al., 2014).

Returning to the molecular mechanisms that mediate the biological cost associated with certain horizontal β–lactamases, and taking into account that they may not be necessarily common to all enzymes, it would be very interesting to fully decipher them in order to better understand the interplay between their dependent resistance and fitness/virulence in *P. aeruginosa*. The knowledge gaps to be filled in this context are mostly related to the intrinsic burden of specific β-lactamases, being of great interest to confirm that globally disseminated enzymes are virtually cost-free unlike specific amino acid variants, that could be associated with great costs, fact determining their limited appearance. In this regard, particularly interesting would be the analysis of fitness costs associated with horizontal β-lactamase variants (mainly OXA-2, OXA-10, and KPC-2/−3 derivatives) that are progressively arising as novel resistance determinants pushed by the selective pressure exerted by recently introduced antipseudomonal drugs such as ceftolozane/tazobactam, ceftazidime/avibactam, and imipenem/relebactam, which have never been studied from the fitness/virulence impact perspective (Fraile-Ribot et al., 2018; Arca-Suárez et al., 2019, 2021; Faccone et al., 2022; Lasarte-Monterrubio et al., 2022; Tu et al., 2022; Yang et al., 2023). Finally, it would also be desirable to ascertain whether or not certain plasmid-encoded enzymes are linked to genes responsible for burden-alleviating mechanisms, which could therefore become interesting therapeutic objectives. Another topic not addressed to date in *P. aeruginosa* is to decipher whether the presence of the acquired β-lactamase gene in the original plasmid vs. its integration in the chromosome (an outcome happening in a considerable number of cases), could entail differential biological costs. All this knowledge yet to be developed in future could help unveil targets aimed at attenuating *P. aeruginosa* virulence and/or hinder the dissemination of certain β-lactamases, thereby aiding to curb the threat posed by this fearsome species.

## 3. Mutation-driven resistance

As will be seen in this section, information concerning the relatedness between mutation-driven mechanisms and fitness/virulence in *P. aeruginosa* (Table 2) is greater than for horizontally-acquired resistance. As mentioned above, although a general idea of decreased fitness/virulence associated with resistance can be obtained mostly from studies with collections of clinical strains (Mulet et al., 2013; Sun et al., 2013; Gómez-Zorrilla et al., 2016; Kaiser et al., 2017), when a deeper approach is performed one can see that several exceptions exist and many clarifications need to be done. Moreover, in this type of studies the resistance phenotype is treated as a whole, i.e., the result of a sum of features, and thus each particular pathway or even a set of different mechanisms for a given antibiotic are not addressed separately. In fact, it is usual, in these investigations, for considerable numbers of clinical strains to display high resistance levels [e.g., Multidrug-Resistant (MDR), Extensively Drug-Resistant (XDR) (Magiorakos et al., 2012)] derived from combining mutation-driven and horizontally-acquired mechanisms, making it very difficult to establish associations between specific resistances and consequences for fitness/virulence. Another confounding factor potentially influencing resistance-virulence balance is the clonal lineage of the strains since, evidently, depending on the initial pathogenic level of each (i.e., strong associations between certain sequence types and the presence of potent virulence factors such as the ExoU toxin), the resistance-associated burden could vary greatly (Hendrickson et al., 2001; Sánchez-Diener et al., 2017; Del Barrio-Tofiño et al., 2020; Recio et al., 2020).

There are other facts revealing the complexity of the topic: for instance, although in some studies several typical virulence-related parameters appear to be impaired in association with high-level resistance (pigment production, motility, lethality in animal models, susceptibility to serum, etc.), some other specific features such as biofilm formation seem to be improved (Mulet et al., 2013; Kaiser et al., 2017). Even some examples of controversial results exist, i.e., data showing XDR strains with increased *in vitro* competitiveness compared to more susceptible ones, demonstrating again the intricate nature of mutational resistance-fitness balance (Kaiser et al., 2017). In any case, since dissecting the knowledge related to wide collections of clinical strains is not a primary objective here, and there are already some reviews addressing the topic (Sawa et al., 2014; Oliver et al., 2015; Juan et al., 2017a), we will focus on basic research only. Moreover, despite fitting with the basic research concept, other studies with *in vitro*-evolved mutants also analyze the final resistance phenotype as a whole and in many cases, several mutations need to be accumulated in order to enable certain resistances (Cabot et al., 2016, 2018; Gomis-Font et al., 2020, 2022; Barceló et al., 2021), making it difficult to extract cause-effect relationships between specific targets and impacts on fitness and virulence. For these reasons, we will mostly approach here the cases of unequivocal interconnection between a specific resistance mechanism and a final impact on fitness/virulence in *P. aeruginosa*, always from a fundamental perspective (Table 2).

### 3.1. AmpC cephalosporinase

This class C β-lactamase is an inducible enzyme under the control of a LysR family transcriptional regulator, AmpR, which depending on which muropeptides coming from the degradation of the peptidoglycan it is bound to, acquires different conformations. Depending on the conformation, AmpR can exert a repressor (in regular conditions) or a transient promotor role of *ampC* expression (during the challenge with an inducer β-lactam) which enables *P. aeruginosa* natural resistance to aminopenicillins and 1st and 2nd generation cephalosporins (Juan et al., 2017b; Torrens et al., 2019a). Besides this regular inducible nature, AmpC stable hyperproduction probably poses the most relevant resistance mechanism in this species, affecting monobactams, antipseudomonal penicillins, and 3rd/4th generation cephalosporins, and can be achieved through the selection of mutations in different targets. For instance, mutational disruptions of the low mass PBP4 (*dacB* gene), the cytosolic amidase AmpD or the cytosolic muropeptide ligase Mpl, are highly prevalent in clinical strains (Del Barrio-Tofiño et al., 2017; López-Causapé et al., 2018; Torrens et al., 2022). These mutations end up altering the peptidoglycan metabolism and the derived pool of muropeptides binding to AmpR, constitutively enabling its activator role. In addition, certain specific amino acid changes in AmpR itself (e.g., D135N or G154R) have been shown to cause its constitutive activator conformation as well (Cabot et al., 2012; Caille et al., 2014; Juan et al., 2017b; Torrens et al., 2019a,b).

As expected, the repressed production of AmpC does not entail any biological cost for *P. aeruginosa* (Pérez-Gallego et al., 2016; Barceló et al., 2022b) and what is more, its deletion caused a decreased fitness in a murine model of infection (Roux et al., 2015). The mechanistic basis for this intriguing apparent contribution of AmpC to the basal virulence of *P. aeruginosa* remains to be investigated. Moreover, when Cabot and colleagues analyzed the capacity of *P. aeruginosa* to develop β-lactam resistance in mutants devoid of their capacity for AmpC hyperproduction (through the disruption of *ampC* or of *ampG,* encoding the permease that enables the entrance of the AmpR-activating muropeptides into the cytosol), although some complex resistance pathways were selected, their associated biological costs were dramatic (Cabot et al., 2018). These results indicate that AmpC hyperproduction is a very efficient and virtually cost-free resistance mechanism in this species which makes the selection of these alternative pathways highly unlikely (Cabot et al., 2018). In fact, most pathways driving to AmpC hyperproduction (at least *dacB* and *ampD* disruptions) have been shown to display no impacts on virulence (Moya et al., 2008; Pérez-Gallego et al., 2016; Torrens et al., 2019b; Barceló et al., 2022b), which is also supported by the high frequency of occurrence of these mechanisms in nature (Del Barrio-Tofiño et al., 2017). However, a previous work in which AmpC hyperproduction was combined with the presence of the acquired PSE-1 β-lactamase showed a dramatic reduction of virulence factors production and mortality in mice, being proposed a derived defect of intercellular signaling as possible explanation for the results (Ramisse et al., 2000; Table 2).

On the other hand, some amino acid changes in the AmpC sequence itself (e.g., T96I, G183D, or ΔG229-E247) combined with hyperproduction have been shown to cause resistance to ceftazidime/avibactam and ceftolozane/tazobactam, but in exchange for this increased spectrum of conferred resistance, there were virtually non-existent added burdens (Barceló et al., 2022b; Table 2). This circumstance poses a worrisome warning, since apparently there is no biological cost-related barrier to avoid the spread of this kind of mutations (Barceló et al., 2022b). Nevertheless, it had been previously shown that resistance to ceftolozane/tazobactam developed *in vitro* in non-hypermutator strains, was due to other combined mutations that determined global pleiotropic effects associated with a severe fitness cost (Cabot et al., 2014). Conversely, in the hypermutator background, *P. aeruginosa* resistance to ceftolozane/tazobactam was associated with the mentioned confluence of circumstances (hyperproduction and structural modification of AmpC), together with many other mutations determining a much lower biological cost compared to the non-mutator background, probably reflecting the increased likelihood of acquiring compensatory mutations (Cabot et al., 2014). This circumstance was later generally seen for other mutation-driven resistance mechanisms (ceftazidime, ciprofloxacin, and meropenem), although with an extraordinary variability in fitness costs depending on the mutants analyzed (Cabot et al., 2016). At any rate, although the mentioned AmpC structural variations (T96I, G183D, or ΔG229-E247) conferring resistance to ceftazidime/avibactam and ceftolozane/tazobactam have been characterized from the fitness cost perspective, many other derivatives involved in resistance to these and other new antipseudomonal drugs remain to be studied in this regard (Arca-Suárez et al., 2020; Fraile-Ribot et al., 2020; Gomis-Font et al., 2022; Ruedas-López et al., 2022; Gomis-Font et al., 2023).

Still related to the AmpC-associated biological cost in *P. aeruginosa,* but in an even more basic research context, certain particular combinations of features such as the triple disruption of AmpD-AmpDh2-AmpDh3 homologs (that entail AmpC de-repression as well as great peptidoglycan recycling alteration), or the over-expression of AmpC from a multicopy plasmid in a peptidoglycan recycling-defective strain (AmpG KO mutant) have been shown to entail drastic attenuations in terms of *G. mellonella* killing (Pérez-Gallego et al., 2016; Torrens et al., 2017). In these specific conditions, several parameters linked to this outcome were also affected, such as growth rate, motility, cytotoxicity, resistance to peptidoglycan-targeting immune proteins, and inter-bacterial competitiveness. These circumstances were supported by transcriptomics, which revealed a reduced expression of protease LasA, phospholipase C, type IV pili (involved in motility), lipopolysaccharide (LPS) biosynthetic pathways, or type III secretion system (T3SS) components (Pérez-Gallego et al., 2016, Torrens et al., 2017; Table 2). Although the mentioned combinations of factors do not occur in nature, it is interesting to understand the underlying mechanisms mediating the virulence attenuation, as this could provide information to unveil therapeutic targets. In this regard, it has been recently demonstrated that in order to obtain these attenuations, an intact enzymatic activity of AmpC is needed (Barceló et al., 2022b) as had been demonstrated for TEM-1 β-lactamase (Gallant et al., 2005), reinforcing the idea of a residual *peptidoglycanase* activity in *P. aeruginosa* AmpC that could be debilitating the cell wall. In fact, a reduction in the amount of peptidoglycan per cell, concordant with a potential partially degraded sacculus, actually occurred in the mentioned conditions, which could be responsible for an increased susceptibility to lysis mediated by osmotic changes and/or immune weapons and thus entailing reduced fitness/virulence (Pérez-Gallego et al., 2016, Torrens et al., 2017). Therefore, this explanation is in accordance with what is mentioned above for OXA-2 derivatives, i.e., AmpC potentially displaying a significant residual capacity to degrade peptidoglycan (Chang et al., 1990; Bishop and Weiner, 1992; Damblon et al., 1995; Rhazi et al., 1999; Urbach et al., 2008; Fernández A. et al., 2012; Pratt, 2016; Barceló et al., 2022a,b), either the sacculus itself or signal muropeptides participating in potential virulence-regulating networks proposed to exist in *P. aeruginosa* and other species (Folkesson et al., 2005; Escobar-Salom et al., 2023).

In this last regard, it is worth highlighting the role of the AmpC transcriptional regulator, AmpR, which apparently has an important repertoire of virulence-related genes under its control (Kong et al., 2005; Balasubramanian et al., 2011, 2012). This fact is in accordance with the proposed existence of networks of muropeptides acting as signals that, once bound to the appropriate transcriptional regulator (AmpR or others), could enable its conformational changes ultimately affecting the expression of certain genes and thus conditioning pathogenic behavior (Folkesson et al., 2005, Escobar-Salom et al., 2023). Therefore, if the pool of muropeptides is altered through a β-lactamase retaining certain peptidase activity, combined or not with another particular event such as the peptidoglycan recycling blockade, it is reasonable to think that the pathogenic response once these fragments bind to the regulator is also modulated (Folkesson et al., 2005; Barceló et al., 2022a,b; Escobar-Salom et al., 2023). Accordingly, it has been shown that certain specific amino acid changes in AmpR (e.g., G154R), responsible for a constitutive AmpC-activating conformation, also contribute to a decreased virulence of the bacterium, which could be related to a repressor role of this particular AmpR variant over certain pathogenesis-related genes (Table 2). However, this reduction does not seem to pose a significant barrier for the dissemination of the ST175 high-risk clone, in which this AmpR mutation is readily detected (Oliver et al., 2015; Sánchez-Diener et al., 2017).

### 3.2. OprD porin

OprD porin-inactivating mutations, a well-known carbapenem resistance pathway, have been found to strongly increase bacterial fitness/virulence in two studies (Skurnik et al., 2013; Roux et al., 2015), probably through different transcriptional and post-transcriptional changes motivated by the absence of this porin. However, in previous works quite the opposite result had been shown, i.e., OprD deletion was associated with a slight decrease in virulence in murine models (Ramisse et al., 2000, Abdelraouf et al., 2011; Table 2). Differences in the model strains used and the various fitness/virulence-related parameters analyzed in each work might well account for these discordant outcomes.

### 3.3. Efflux pumps

Besides AmpC, efflux pumps are probably the most well-known resistance mechanism of *P. aeruginosa*, with those within the Resistance-Nodulation-Division (RND) family as the most clinically relevant. These pumps are structured in three compounds: an inner membrane transporter, a periplasmic adaptor, and an outer membrane channel, usually encoded as operons. The existence of a connection between certain RND pumps and virulence is a well-known fact in Gram-negative bacteria (Fernando and Kumar, 2013; Rampioni et al., 2017), entailing an obvious interplay with resistance. Regardless, although up to 12 different RND efflux systems have been found in the PAO1 reference strain, the most relevant variants by far in terms of resistance conferred are MexAB-OprM, MexXY-OprM, MexCD-OprJ, and MexEF-OprN (Lorusso et al., 2022). Altogether, they affect virtually all antibiotic families and, similar to AmpC, often display a basal level of expression partially accounting for the intrinsic level of resistance to several drugs, which can be amplified through the selection of mutations that trigger their overexpression. The array of targets/mutations that determine different levels of hyperexpression of these pumps and consequent resistance phenotypes is wide and complex, but we will only approach the specific mechanisms that have been studied from the fitness/virulence point of view. As will be seen here, the topic of relatedness between efflux pumps and virulence, which is intimately linked to quorum sensing, is probably the one with the most published data within the field of resistance-fitness interplay in *P. aeruginosa*, revealing a great complexity that deserves delving into (Table 2).

The MexAB-OprM pump hyperexpression (classically known as *nalB* phenotype) has been related to mutations in different targets such as its repressors *mexR*, *nalC,* and *nalD*, and contributes to resistance to most antipseudomonal antibiotics, especially quinolones and β-lactams (Lorusso et al., 2022). More than two decades ago some studies started to characterize the potential costs associated with the hyperexpression of this pump, suggesting a significant burden for the strains showing this phenotype, with reduced production of pyocyanin, protease and elastase, a slightly impaired capacity to invade cell cultures as well as a significantly attenuated virulence in the nematode *Caenorhabditis elegans* and murine systemic infection models (Evans et al., 1998; Hirakata et al., 2002; Sánchez et al., 2002). The excessive extrusion, driven by the hyperexpressed pump, of some quorum-sensing autoinducers ultimately regulating the expression of virulence genes, was suggested as a possible explanation for these results (Evans et al., 1998; Sánchez et al., 2002). More recent works have also demonstrated a slight cost associated with MexAB-OprM hyperproduction (specifically mediated by *mexR* deletion) in mice, which was very significantly increased when combined with OprD inactivation (Abdelraouf et al., 2011). Moreover, hyperexpression of this efflux pump seems essential to provide resistance to the recently introduced cefepime/zidebactam combination in addition to other mutations affecting PBPs, altogether determining very high biological costs for *P. aeruginosa* (Barceló et al., 2021).

Conversely, other investigations showed that MexAB-OprM hyperproduction (achieved through mutations in *mexR* or *nalD*) was associated with increased virulence in murine models in a strain-dependent fashion: whereas PAO1 mutants displayed a hypervirulent behavior, the pump hyperproduction had no effect on PA14 virulence (Jorth et al., 2017; Vaillancourt et al., 2021). These data, added to those in other studies showing that the deletion/inhibition of *mexAB* and/or *oprM* resulted in dramatically reduced cell invasiveness and drastically impaired fitness/virulence in murine colonization/infection models (Hirakata et al., 2002, 2009; Aoki et al., 2004; Roux et al., 2015), suggest the existence of a very intricate connection between MexAB-OprM-dependent resistance and virulence. Some explanations for these certainly complex results can be provided, such as the suggested contribution of this pump to toxin and/or quorum-sensing signal secretion (Evans et al., 1998; Hirakata et al., 2002; Sánchez et al., 2002; Jorth et al., 2017), the unequal effects of the different types of mutations in *mexR* studied [protein disruption (Jorth et al., 2017) vs. specific amino acid changes (Sánchez et al., 2002)], or the intrinsic features of each *P. aeruginosa* strain used. Either way, all these facts warrant the need for more research to fully understand the influence of MexAB-OprM on the modulation of *P. aeruginosa* fitness/virulence.

The MexCD-OprJ efflux pump is controlled by the repressor NfxB and its regular expression has an apparent low impact on the basal phenotype of *P. aeruginosa* resistance (Lorusso et al., 2022). Several types of mutations in *nfxB* have been described as a cause of MexCD-OprJ increased expression, entailing clinically relevant levels of resistance mainly to quinolones and cefepime (Jeannot et al., 2008; Lorusso et al., 2022). Dealing with virulence interplays, deletion of this pump has no effects on *P. aeruginosa* lethality in a murine model (Hirakata et al., 2002), and in contrast with MexAB-OprM in which intricate results certainly do exist, all studies point in the same direction: overexpression of MexCD-OprJ is associated with significant impairments in fitness/virulence. These attenuations have been shown to be reflected in different features of the bacterium such as growth rate, motility, production of extracellular factors (rhamnolipid, elastase, phospholipase C, and pyocyanin), T3SS activation levels, and resistance to complement system (Linares et al., 2005; Jeannot et al., 2008; Stickland et al., 2010; Martínez-Ramos et al., 2014). The underlying basis for these results seems not to be linked to the effects of the NfxB regulator mutation *per se*, but rather to the inappropriate overexpression of a functional MexCD-OprJ efflux pump triggering pleiotropic changes that globally dysregulate the bacterial physiology (Stickland et al., 2010). Moreover, it has been also shown that, similarly to what has been explained for MexAB-OprM, hyperexpression of MexCD-OprJ caused an excessive extrusion of certain quorum-sensing-related signals (entailing a consequently insufficient intracellular concentration), finally impacting the expression of certain virulence factors controlled by this system (Alcalde-Rico et al., 2018). What is intriguing regarding all these facts is how the hyperproduction of this pump can be selected in clinical strains with a relatively high frequency (Lorusso et al., 2022) if the associated costs are apparently that high? A frequent appearance of compensatory mutations is likely the most logical explanation, but this possibility has not been experimentally ascertained yet.

Basal expression of the MexEF-OprN system does not contribute to the intrinsic resistance of *P. aeruginosa*, but when hyperproduced it significantly increases quinolone resistance levels. Regulation of MexEF-OprN is more complex than the rest of pumps, with it being influenced by different elements such as MexT (a LysR-like transcriptional activator), the oxidoreductase MexS, and the ParRS two component system, among others (Wang et al., 2013; Lorusso et al., 2022). Hyperexpression of MexEF-OprN is often associated with a concomitant decreased expression of the porin OprD (and derived carbapenem resistance), which is based on the fact that MexT, besides regulating MexEF-OprN expression, also acts as an *oprD* repressor. Thus, amino acid changes driving to increased activity of MexT regarding both roles would explain this phenomenon (Lorusso et al., 2022). Since, as mentioned above, OprD loss seems to be related to increased fitness and virulence (Skurnik et al., 2013; Roux et al., 2015), it would be expectable that decreased expression of this porin appearing in parallel to MexEF-OprN hyperexpression would have similar effects, which could be partially alleviating the attenuation associated with the pump. However, this possibility has not been experimentally demonstrated yet.

Returning to MexEF-OprN itself, it was proposed more than two decades ago that hyperproduction of this pump involved an excessive extrusion of certain quorum-sensing signals that would finally cause a reduced production of pyocyanin, elastase, and rhamnolipids (essential for correct swarming motility), all under the control of the *las* and *rhl* systems of *P. aeruginosa* (Köhler et al., 2001). Decreased activity of T3SS was also seen to be associated with MexEF-OprN overexpression (Linares et al., 2005), which on the other hand was shown to cause several changes in the bacterial transcriptome, principally in terms of reduced expression of quorum-sensing regulated genes. These changes, which entailed significant virulence attenuations in different infection models (*Dictyostelium discoideum, C. elegans*, rodents), were found to be pump-dependent as opposed to MexT-dependent (Cosson et al., 2002; Lamarche and Déziel, 2011; Olivares et al., 2012). However, MexT was also reported to display a direct capacity of control over other virulence-related features such as the expression of components of T3SS in a MexEF-OprN-independent fashion, reinforcing the complexity of the topic (Tian et al., 2009). It was later shown that the biological burden associated with MexEF-OprN, and in fact with the hyperproduction of the rest of the pumps, is partially alleviated through a metabolic re-accommodation based on an increased expression of the anaerobic nitrate respiratory chain, a response envisaged as an interesting therapeutic target (Olivares et al., 2014; Olivares Pacheco et al., 2017). Finally, in accordance with the burden associated with hyperproduction, the contrary situation (i.e., deletion of the pump or, what is virtually the same, inactivation of MexT) has been shown to increase *P. aeruginosa* virulence *in vivo*, a feature at least partially based on a documented increase in collagenase activity, rhamnolipid production, and swarming motility (Olivas et al., 2012; Wang et al., 2013; Luong et al., 2014; Vaillancourt et al., 2021). However, the underlying mechanisms explaining these results remain to be investigated.

Finally, although MexXY-OprM is the typical pump, in some strains the MexXY operon contains a coding sequence for another outer membrane protein, OprA, which provides the MexXY-OprA variant. Regardless, this antimicrobial-inducible pump displays quite a wide array of affected antibiotics, among which aminoglycosides are probably the most efficiently extruded. Different mutations driving to the hyperproduction of this pump have been described, but few studies have addressed the associated biological costs (Lorusso et al., 2022). In this regard, it was shown two decades ago that a *P. aeruginosa* mutant defective in MexXY displayed partially decreased invasiveness and lethality in a murine model (Hirakata et al., 2002). Conversely, impaired pyoverdine production associated with MexXY-OprM hyperproduction has recently been reported (Ikarashi et al., 2021), suggesting that only an appropriate level of expression of this pump enables a regular pathogenic behavior. These facts once more evidence the complex interplay between efflux pumps and the modulation of virulence in *P. aeruginosa*.

Finally, although their impact on *P. aeruginosa* antibiotic resistance seems marginal, other efflux pumps have been studied from the fitness/virulence point of view. In this regard, the alteration of certain components of the MexGHI-OpmD pump resulted in a marked reduction in the production of specific quorum-sensing signals and caused the attenuation of virulence in rat and plant models (Aendekerk et al., 2005). Thus, this pump seems to be intimately related to quorum-sensing (Karatuna and Yagci, 2010), a general feature for all the aforementioned ones (perhaps with the only exception of MexXY), but it has also been linked to the delivery of a phenazine required for correct biofilm formation which would have obvious virulence-related implications (Sakhtah et al., 2016). On the other hand, inactivation of the MuxABC-OpmB system, which causes an increased level of carbenicillin resistance, also entailed significant impairments in motility and virulence in animal/plant models. However, the underlying mechanisms and biological relevance of this phenomenon have not been delved into (Yang et al., 2011).

### 3.4. DNA gyrase/topoisomerase IV

These two enzymes (each comprising two subunits: GyrA and GyrB, and ParC and ParE, respectively) are involved in the DNA replication process by modulating the level of its supercoiling through their nuclease/ligase activities. These enzymes are the target of quinolones, which form complexes with them, thus disabling their regular activity, thereby blocking DNA replication and finally promoting its fragmentation and derived cell death (Malik et al., 2006; Bruchmann et al., 2013). These type 2 topoisomerases pose a paradigm of mutation-driven resistance caused by alteration of the target and derived loss of affinity for the drug. Many amino acid changes in their sequences have been demonstrated to cause different levels of quinolone resistance in *P. aeruginosa* and other species, which justifies the denomination of certain positions within their sequences as Quinolone Resistance-Determining Regions (QRDR). Interestingly, not all the mutations in the QRDR have the same effect on the derived resistance level, and as could be expected, there is an accumulative effect in terms of the phenotype conferred when sequential changes in QRDR are selected over time (Bruchmann et al., 2013). In this regard, mutations in *gyrA* and *parC* are probably those most usually found in *P. aeruginosa* clinical strains (nearly universal in high-risk clones), and are usually combined with a concomitant hyperexpression of efflux pumps (Bruchmann et al., 2013; Del Barrio-Tofiño et al., 2017; Melnyk et al., 2017). In terms of biological costs associated with QRDR mutations in *P. aeruginosa*, there is a surprisingly low number of publications in the field of basic research (Table 2), with that of Kugelberg and colleagues as the pioneer work (Kugelberg et al., 2005). In this study, low level resistance was shown to be readily obtained *in vitro* through different single mutations in *gyrA* or *gyrB*, which in the vast majority of cases were cost-free. However, when selecting mutants in intermediate concentrations of norfloxacin (8 mg/L), some of them were associated with very important growth impairments. This was a general feature for all the selected high level resistant mutants, which always displayed two mutations: either in two topoisomerase genes or in one topoisomerase gene plus *nfxB*. The biological cost of these mutations was related to a partial loss of function of the mutated protein(s): a decreased supercoiling of DNA was appreciated in the strains with reduced fitness, which would in turn dampen DNA transcription/replication thereby reducing growth rates. When the appearance of compensatory mutations was studied, it was seen that, due to them, the supercoiling level was recovered in parallel to the regular growth rate (although a slight decrease in quinolone resistance level was also reported) (Kugelberg et al., 2005). In any case, what can be deduced from this study and from the epidemiological panorama of quinolone-resistant *P. aeruginosa* clinical strains nowadays (Oliver et al., 2015; Kocsis et al., 2021) is that, although some mutations may entail an important fitness cost, selection of compensatory mutations seems a frequent outcome that suggests that this type of resistance is not an unbearable handicap. In this regard, in a more recent work (Melnyk et al., 2017) it was shown that the appearance of cost-free quinolone-resistant mutants was much more likely under a discontinuous regime of ciprofloxacin compared to a constant one, and that the main driver for this output was the appearance of second-site compensatory mutations rather than the initial selection of cost-free alleles. However, in this study, virtually all the mutants obtained displayed combined mutations in *nfxB* plus *gyrA* or *gyrB*, making it difficult to draw mechanism-specific conclusions (Melnyk et al., 2017).

Finally, regardless of compensatory mutations, a fact that likely contributes to the success of quinolone-resistance in *P. aeruginosa* is the well-known association between the prevalence of mutations in QRDR and the presence of the T3SS-exported ExoU toxin (highly cytotoxic and whose codification is usually exclusive for the less virulence-conferring ExoS) (Wong-Beringer et al., 2008; Agnello and Wong-Beringer, 2012; Cho et al., 2014). This circumstance could be interpreted as an additional strategy to minimize the burden associated with quinolone resistance; that is to say, if the initial level of virulence of a strain is very high (like that of ExoU-positive strains), the burden associated with resistance mechanisms would have less impact than if the original virulence was lower. However, some recent data suggest that the presence of ExoU *per se* also determines direct changes in *P. aeruginosa* biology enabling a better regulation of DNA supercoiling, which alleviates the burden linked to topoisomerases mutations (Agnello et al., 2016). Therefore, from this last study one could deduce that not only can resistance mechanisms have a direct impact on fitness/virulence, but also the opposite: the presence of certain virulence factors, such as ExoU, could influence the dynamics of mutation-driven resistance acquisition by finely tuning the associated costs. This is a groundbreaking idea that should be delved into, since other unknown phenomena of virulence factors ultimately determining the fate of different mutational resistance pathways acquisition may exist, which could pose interesting interconnections to be exploited from the therapeutic point of view.

### 3.5. LPS modification

LPS modifications consisting of the addition of positively charged molecules are the major polymyxin resistance mechanism in *P. aeruginosa*, and can be achieved through different pathways driving to a reduction in the affinity of the drug. The aminoarabinosylation of LPS lipid A has been described as the primary colistin (polymyxin E) resistance mechanism in *P. aeruginosa*, and is usually associated with mutations in different two-component systems (e.g., PhoPQ, ParRS, CprRS, or PmrAB) leading to the overexpression of the *arnBCADTEF* operon, which encodes the enzymes that perform the addition of 4-amino-4-deoxy-l-arabinose to lipid A (Jeannot et al., 2017). Regarding the biological costs of this mechanism, it has been shown that the overexpression of this operon and the derived aminoarabinosylation have virtually innocuous effects for the fitness and virulence of *P. aeruginosa* (Lo Sciuto et al., 2020). In addition, in another work analyzing the collateral effects of resistant mutants obtained in a morbidostat device, although an increased serum resistance and biofilm formation capacity appeared in parallel to colistin resistance, virulence attenuation in *G. mellonella* model was also seen after 21 days of exposure to the drug, once more evidencing the intricate nature of resistance vs. fitness/virulence interplay (Dößelmann et al., 2017; Javed et al., 2021). Finally, another less known potential colistin resistance mechanism, namely the addition of phosphoethanolamine to lipid A through the chromosomal enzyme EptA (Nowicki et al., 2015; Freire et al., 2021), has also been approached regarding its potentially associated burden. In this sense, EptA hyperexpression has been shown to cause growth, competitiveness, and cell envelope defects that were not linked to phosphoethanolamine transferase activity *per se* (Cervoni et al., 2023). This fact could explain why other mechanisms based on aminoarabinosylation are those appearing in clinical strains and *in vitro* evolution experiments (Cervoni et al., 2023; Table 2).

### 3.6. Other mechanisms

Finally, some other specific acquired/intrinsic resistance mechanisms have been assessed in terms of their associated costs, and whereas fosfomycin resistance achieved through deletion of *glpT* (encoding for the glycerol-3-phosphate transporter that enables this drug uptake) caused no measurable fitness defects or even a hypervirulent response (Castañeda-García et al., 2009; Rodríguez-Rojas et al., 2010; Roux et al., 2015), the deletion of *aph* (encoding for an aminoglycoside phosphotransferase for high-level resistance to kanamycin) caused decreased virulence (Roux et al., 2015). Therefore, these data demonstrate once more that cases in which antimicrobial resistance mechanisms are devoid of costs or even contribute to pathogenesis are not exceptional in *P. aeruginosa*, and thus contradict the general notion accepting that resistance always entails a fitness cost.

## 4. Concluding remarks and future directions

As shown throughout this review, the topic of the balance between resistance and fitness/virulence in *P. aeruginosa* in the field of basic research applied to the context of acute infection has been significantly explored, providing interesting data revealing a great complexity. The published information is especially abundant in the case of mutation-driven mechanisms, and in this specific field but also in that of horizontally-acquired resistance, the diversity of data is astonishing, even with a considerable number of contradictory results. The information gathered here also evidences that there are still many knowledge gaps that need filling, for instance regarding most of the horizontally acquired β-lactamases and aminoglycoside-modifying enzymes, as well as specific mutation-driven mechanisms such as, among others, mutations in the PBP3 encoding gene (*ftsI*) or in elongation factor G (*fusA1*), triggering aztreonam and aminoglycoside resistance, respectively. These mechanisms, despite being reported relatively often in clinical strains, have been barely studied in terms of their associated costs (Jorth et al., 2017; Bolard et al., 2018; McLean et al., 2019).

Another field that has been barely approached is the balance between resistance and fitness/virulence not from an acute infection perspective as we have done, but in the context of chronic infection, in which the concept of fitness is much more complex. That is to say, selection for slow growth (which enables a better adaptation to nutrient scarcity and resistance to certain antibiotics) and reduced virulence (triggering a less effective activation of the immune system) is a typical outcome of *P. aeruginosa* in cystic fibrosis infections for instance (Høiby et al., 2010; Lorè et al., 2012; Malhotra et al., 2019), and therefore, the fitness burden assumed to be negative in the acute context, seems that could be somehow advantageous in the chronic one. In fact, there are very few investigations specifically assessing the antibiotic resistance burden applied to chronic infection (McCaughey et al., 2013; Rundell et al., 2020). Hence, research delving into this intricate field is warranted to better understand the complex adaptive needs that *P. aeruginosa* have to cope with in such a particular niche.

Returning to the acute context, gaining basic knowledge about resistance-fitness/virulence interplay is envisaged as an essential weapon to reveal therapeutic targets, especially if we consider that new antipseudomonal drugs have been introduced in the last few years (ceftolozane/tazobactam, imipenem/relebactam, ceftazidime/avibactam, and cefiderocol) and others (aztreonam/avibactam, cefepime/zidebactam, and cefepime/taniborbactam) are likely to be approved in the near future (Yahav et al., 2020). Therefore, we need to determine whether the resistance mechanisms selected against these new drugs could inevitably carry high biological costs, and consequently entail increased difficulties for mutation-driven compensation. Obviously, information regarding the fitness cost for these new drugs is still scarce (Gomis-Font et al., 2020; Barceló et al., 2021) and must be amplified since it may be useful for the development of anti-virulence and/or resistance-breaking strategies (Mark et al., 2011; Guillard et al., 2016; De Oliveira et al., 2020; Rezzoagli et al., 2020; Langendonk et al., 2021; Liao et al., 2022; Reig et al., 2022). Unfortunately, the analysis of the epidemiological picture of the resistance phenomenon in *P. aeruginosa* reveals a warning signal as is the clear association between certain mutation-driven/horizontal mechanisms and successful high-risk clones, indicating that the associated burden of classic antibiotic resistance is not a significant barrier to the appearance and dissemination of many mechanisms (Oliver et al., 2015; Del Barrio-Tofiño et al., 2020; Kocsis et al., 2021). This idea has to be considered as a real possibility also for the mentioned new antipseudomonal agents [e.g., AmpC/FOX variants causing ceftolozane-tazobactam resistance with very little burden, see above (Barceló et al., 2022b)], but still, a deeper analysis must be approached with the hope of finding new drugs (including combinations with classic ones) for which resistance mechanisms are necessarily associated with high fitness/virulence impairments. Finally, since it is a topic in which research is not yet especially prolific, a more profound analysis of *P. aeruginosa* compensatory evolution associated with the different resistance mechanisms (especially regarding the most recent drugs) is needed in order to identify the genes responsible for compensation mechanisms and determine whether they could be used as alternative therapeutic targets.

Beyond the field of drug development, complete insight into the topic in basic research terms could make it easier to decipher the intricate picture of resistance-virulence co-evolution in the clinical environment. In other words, it could help to understand how antibiotic resistance, depending on the specific mechanism(s), may tune the virulence of *P. aeruginosa,* thus enabling a prediction of the evolution of pathogenesis and consequences for the patient, which could be very useful in clinical practice (Geisinger and Isberg, 2017). For this purpose, other factors that should be considered, and that are addressed in other reviews are: type of infection, patient background, lineage of the *P. aeruginosa* strain and its arsenal of virulence factors, and the nature of antibiotic treatments received (Mulet et al., 2013; Peña et al., 2015; Sánchez-Diener et al., 2017; Juan et al., 2017a). If we manage to integrate all these parameters to fully understand the resistance-fitness balance in *P. aeruginosa*, we will hopefully be capable of predicting which treatments could be more likely to cause deleterious biological costs associated with resistance development, with obvious positive implications for the patients. Unfortunately, these goals still seem far off for the time being and in any case, in order to attain them it is necessary to fully understand the elementary bases that govern resistance-virulence interplay in *P. aeruginosa*, the field addressed in this review.

A final topic that has been barely studied in specific terms is the fitness cost potentially associated with the phenomenon of adaptive resistance in *P. aeruginosa*. Given the close relation of this type of transient resistance with stress responses, two-component systems and quorum sensing signaling (Gooderham and Hancock, 2009; Fernández L. et al., 2012; Stewart et al., 2015; Sandoval-Motta and Aldana, 2016; Moradali et al., 2017; Ducret et al., 2022), along with the well-known influence that most of these elements exert on virulence, a connection between adaptive resistance and the temporary modulation of pathogenic behavior is highly likely (Strempel et al., 2013). Thus, this is a virtually virgin topic that should be delved into with the goal of delineating therapeutic strategies to disable this type of inducible resistance and/or take advantage of the potential associated costs.

Altogether, the data reviewed here suggest that we need a holistic approach to understand all the edges of the interplay between resistance and fitness/virulence in *P. aeruginosa*, including basic but also clinical points of view. Opening/delving into new fields of research such as those mentioned in this manuscript is a must in order to defeat one of the greatest challenges for public health in the twenty-first century, as is *P. aeruginosa* antibiotic resistance.

## Author contributions

EJ-L: Investigation, Writing – original draft. IB: Investigation, Writing – original draft. ME-S: Investigation, Writing – original draft. ME: Investigation, Writing – original draft. LZ: Conceptualization, Supervision, Validation, Writing – original draft. SG-Z: Conceptualization, Funding acquisition, Supervision, Validation, Writing – original draft. ES: Conceptualization, Supervision, Validation, Writing – original draft. AO: Funding acquisition, Supervision, Validation, Writing – review & editing. CJ: Conceptualization, Funding acquisition, Supervision, Validation, Writing – review & editing.

## Funding

The author(s) declare financial support was received for the research, authorship, and/or publication of this article. This work was supported by the Balearic Islands Government grant FPI/2206/2019 and grants IJC2019-038836-I (*Ministerio de Ciencia e Innovación*, Spain), PI21/00509, PI21/00753, PI21/00017, FI19/00004 and CB21/13/00099 [*Centro de Investigación Biomédica en Red-Enfermedades Infecciosas* (CIBERINFEC-ISCIII)] from the *Instituto de Salud Carlos III* (Spain) co-financed by the European Regional Development Fund “A way to achieve Europe” and the contract CM22/00008 funded by Instituto de Salud Carlos III and cofunded by the European Union – NextGenerationEU.

## Conflict of interest

The authors declare that the research was conducted in the absence of any commercial or financial relationships that could be construed as a potential conflict of interest.

## Publisher’s note

All claims expressed in this article are solely those of the authors and do not necessarily represent those of their affiliated organizations, or those of the publisher, the editors and the reviewers. Any product that may be evaluated in this article, or claim that may be made by its manufacturer, is not guaranteed or endorsed by the publisher.
